# Distribution of Cadmium in Fresh Vegetables Marketed in Southeast China and Its Dietary Exposure Assessment

**DOI:** 10.3390/foods12061204

**Published:** 2023-03-12

**Authors:** Xiao-Dong Pan, Jian-Long Han

**Affiliations:** Zhejiang Provincial Center for Disease Control and Prevention, Hangzhou 310051, China; xdpan@cdc.zj.cn

**Keywords:** vegetables, cadmium, toxic metals, dietary exposure, risk assessment

## Abstract

This study investigated concentrations of cadmium (Cd) in 2465 vegetable samples (52 species) from 2018 to 2022 and estimated the associated health risk for local consumers. The average concentration of Cd was 0.035 mg kg^−1^, and the percentage of samples exceeding the Chinese maximum allowed concentration was 3.89% (96/2465). The top five species with highest Cd levels were *Lilium brownii* F (0.182 mg kg^−1^), *Allium chinense* G (0.117 mg kg^−1^), *Allium macrostemon* Bunge (0.105 mg kg^−1^), *Colocasia esculenta* (0.064 mg kg^−1^), and *Amaranthus tricolor* L (0.054 mg kg^−1^). Bulb vegetables had a higher relative accumulation of Cd compared to other vegetables. The levels of Cd in vegetables varied significantly across sampling areas and years. The mean estimated daily intake (EDI) of cadmium through consumption of vegetables was 0.519 μg kg^−1^ bw per day for adults and 0.217 μg kg^−1^ bw per day for children. The target hazard quotients (THQs) were all less than the threshold of 1 for both adults and children. This indicates that there is low health risk for Cd through vegetable consumption. However, routine monitoring of Cd levels in food is still crucial to ensure food safety and protect public health.

## 1. Introduction

Vegetables are important sources of food for the world’s population. In Asia, especially in China, they account for a large proportion of the food structure due to the traditional vegetarian habit. The Dietary Guidelines for Chinese Residents (2016 Edition) suggest that the daily vegetable intake for the general population should be between 300 and 500 g [1]. Vegetable consumption can provide carbohydrates, protein, dietary fiber, vitamins, and minerals to the human body. However, there is increasing concern about the accumulation of toxic substances from the environment.

Toxic metal pollution is a worldwide environmental problem that causes tremendous harm to public health. Soil can easily become polluted with metals due to industrial wastewater, mining emission, fossil fuel burning, sewage sludge, and fertilizers [2]. Once these chemicals enter the soil and water, they can be absorbed by plants and transferred to the human body through the food chain. According to a nationwide survey by the Chinese Ministry of Environmental Protection (MEP) [3], 19.4% of recognized cropland locations had accumulated toxic metals at abnormal levels. Cadmium (Cd) is the main pollutant, with 7% of soil samples exceeding the Chinese soil quality limit [4].

Cd has been widely used in metal electroplating industries, batteries, ceramics, electronic instruments, pigments, petroleum products, textiles, insecticides, solders, synthetic chemicals, and photography [5]. When Cd accumulates in the human body, it can cause irreversible damage to biological systems. Furthermore, the Cd in the human body is usually bound firmly to metallothioneins [6]. The liver and kidney are the main targets of Cd, which has a long biological half-life, about 2–3 decades in the kidney [7]. Chronic exposure to elevated levels of Cd can cause liver damage, bone degeneration, blood damage, and renal dysfunction.

Several reports have revealed [8] varying concentrations of Cd in different types of vegetables. For instance, Singh et al. discovered 5.35 mg kg^−1^ dry weight (dw) of Cd in *B. vulgaris* from carpet industrial and residential areas in India. Ahmed et al. [9] observed 0.19–0.83 mg kg^−1^ dw of Cd in leafy vegetables, such as spinach, water spinach, malabar spinach, jute mallows, red amaranths, and stem amaranths from the Keraniganj agricultural area in Bangladesh. Quispe et al. [10] found 0.0136 mg kg^−1^ wet weight (ww) of Cd in peppermint, coriander, garlic, and leek from Arequipa, Peru. Chen et al. [11] reported an average value of 0.17 mg kg^−1^ ww of Cd in vegetables, where 34.3% of leafy vegetables and 33.3% of rootstalk vegetables exceeded the Chinese maximum permissible limit (0.02 mg kg^−1^ ww for leafy vegetables and 0.1 mg kg^−1^ ww for rootstalk and legume vegetables) in Xiangtan county, Hunan province, south China.

The Yangtze River Delta region in southeast China has high population density and rapid economic activities. Our previous reports [12,13,14,15,16,17] have found potential pollution of toxic metals in marine fish, fresh meat, rice and seaweeds in the region. Accordingly, it is reasonable to hypothesize that there is potential metal contamination in vegetables from the Yangtze River Delta region in southeast China. In this study, we conducted an extensive investigation on the concentration of cadmium (Cd) in vegetables by collecting a large number of samples from Zhejiang province in southeastern China. The characteristics of Cd distribution and target hazard quotient (THQ) for local residents were analyzed. The data from this study will provide scientific support for population health risk assessment in this area and inform future efforts to monitor and regulate the levels of cadmium and other toxic metals in food.

Overall, the study underscores the importance of ongoing research and monitoring efforts to ensure food safety and to protect public health in regions with high levels of economic activity and population density. By identifying and addressing potential sources of pollution and contamination, researchers and policymakers can work together to promote a safe and healthy food supply for all.

## 2. Materials and Methods

### 2.1. Monitoring Samples

**Figure 1:**
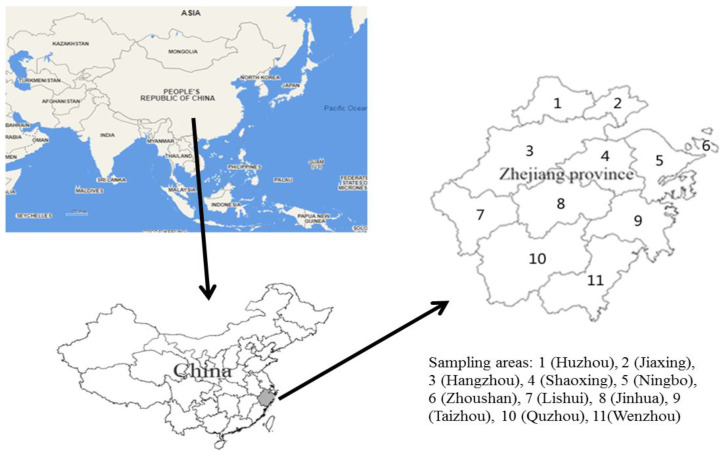
The simple map of sampling areas in Zhejiang province, southeast China.

From 2018 to 2022, 2465 fresh vegetable samples were collected from 11 monitoring areas in Zhejiang Province. The simple sampling map is shown in Figure 1. The simple map was drawn by MapGIS K9 SP2 free trial edition (Zondy Cyber Comp., Beijing, China). The analysis of monitoring samples was completed by the laboratories of the Centers for Disease Control and Prevention (CDC) in Hangzhou, Huzhou, Jiaxing, Jinhua, Lishui, Ningbo, Quzhou, Shaoxing, Taizhou, Wenzhou and Zhoushan. All edible parts of samples were collected and homogenized, and they were pretreated and tested immediately within one day.

In totaL., 2465 fresh vegetable samples were classified into 10 types with 52 species. They were Brassica (*Brassica campestris* L., *broccoli*, cabbage, cauliflower and purple cabbage), bulb vegetable (*Allium cepa* L., *Allium chinense* G., *Allium macrostemon* Bunge, *Allium tuberosum* Rottler, *Lilium brownii* F.), Cucurbitaceae (*Benincasa hispida*, *Cucumis sativus* L., *Cucurbita moschata*, *Cucurbita pepo*, *Lagenaria siceraria*, *Lagenaria siceraria var*. hispida, *Luffa aegyptiaca* Miller, *Momordica charantia* L.), leaf vegetable (*Amaranthus tricolor* L., *Apium graveolens* L., *Brassica campestris* L., *Brassica rapa var.* glabra RegeL., *Coriandrum sativum* L., *Glebionis coronaria*, *Houttuynia cordata*, *Ipomoea aquatica* Forsk, *Lactuca sativa* L., *Lactuca sativa var* longifoliaf, *Spinacia oleracea* L.), stem vegetable (*Asparagus officinalis* L., *Apium graveolens* L., *Lactuca sativa var* angustana), fresh legume vegetable (*Phaseolus vulgaris* L., *Vigna unguiculata*), Aquatic vegetable (*Artemisia selengensis* Turcz, *Eleocharis dulcis*, lotus root, *Oenanthe javanica*, *Trapa bispinosa* Roxb, *Zizania latifolia*), root and tuber vegetable (*Colocasia esculenta*, *Daucus carota*, *Dioscorea polystachya*, *Ipomoea batatas*, *Raphanus sativus* L., *Solanum tuberosum* L., *Zingiber officinale* Roscoe), Solanaceous vegetables (*Capsicum annuum* L., green pepper, *Solanum lycopersicum* L., *Solanum melongena* L.), and bamboo shoot. The samples were stored at −20 °C.

### 2.2. Analysis and Quality Control

The concentrations of Cd in vegetables were analyzed using the Chinese standard analysis method of GB 5009.268-2016 [18]. Briefly, fresh samples (1–2.0 g) were digested in acid-clean Teflon vessels containing 8 mL HNO_3_ in a Mars-6 microwave digestion system (CEM, Charlotte, NC, USA). The samples in closed vessels were heated at 190 °C for 20 min. After digestion, the residue was heated at 150 °C until nearly dry. Then, it was diluted to 20 mL by de-ionized water and analyzed by the ICP-MS instrument (NexION 300, Perkin Elmer, Inc., Shelton, CT, USA). To ensure the quality of sample analysis, the certified reference materials (CRMs) (GBW10014 cabbage and GBW10015 spinach) were analyzed coupled with samples. The Cd values of CRMs were tested alongside the samples, and the results were within 10% of certified values (0.035 ± 0.006 and 0.019 ± 0.02 mg kg^−1^). Furthermore, the spiking samples (blank sample matrix addition of 0.05 mg kg^−1^ to blank sample matrix) were investigated for quality control. All recoveries of spiking ranged from 90% to 105%. The limit of detection (LOD) was defined by three times the standard deviation of 10 runs of blank measurements. The LOD of Cd was found to be 0.002 mg kg^−1^.

### 2.3. Exposure Assessment

All test data were processed according to the Assessment Method for Low Content Pollutants in Food stipulated by the WHO in 1995 [19]. That is, when the number of undetected samples is not more than 60% of the total number, the undetected value is expressed as half of the LOD.

The target hazard quotient (THQ) was used to assess non-carcinogenic risk of cadmium intake by residents in vegetables [20]. THQ is calculated as follows, THQ = EDI/RfD. Wherein, RfD is the reference dose of pollutants, referring to the recommended monthly cadmium tolerance dose of JECFA PTMI 0.025 mg kg^−1^ body weight (bw) (equivalent to 0.8 μg kg^−1^ bw per day). EDI is the daily metal intake caused by eating vegetables. EDI is calculated as follows: EDI = (R × C)/BW, where R is the vegetable consumption. According to the data of the dietary survey report of Zhejiang Province, adults take 273.30 g d^−1^ and children take 185.7 g d^−1^; C is the concentration of cadmium; BW is the average weight of adults (18 years old and above), 60 kg, and children (7–18 years old), 30 kg.

For cancer risk analysis, the lifetime target cancer risk (TR) was calculated by multiplying the daily dose by the cancer slope factor (CSF) derived from the response–dose curve for toxicant ingestion. TR is calculated as: TR = EDI × CSF × 10^−3^. Based on the USEPA Integrated Risk Information System (USEPA, 2010) database, the CSF value for Cd is 6.3 mg kg^−1^ per day. If TR > 1 × 10^−4^, it indicates that the risk of developing cancer over a human lifetime is a 1 in 10,000 carcinogenic risk. If 1 × 10^−6^ < TR < 1 × 10^−4^, it indicates an acceptable level of carcinogenic risk. If TR < 1 × 10^−6^, it indicates a negligible carcinogenic risk [10].

### 2.4. Data Processing and Statistical Analysis

SAS JMP 10.0 (free trial version) software was used for statistical analysis, and Mapinfo 12.0 (free trial version) software was used to draw the geographic spatial distribution map. The data of various toxic metals cadmium were tested by Shapiro–Wilk W, and the results showed that the data were presented as a non-normal distribution. Therefore, Kruskal–Wallis test in the non-parametric test method was used to analyze the statistical difference of Cd content among different areas and years. An alpha level of 0.05 was adopted for all statistical groups.

## 3. Results and Discussion

### 3.1. Cadmium Content in Vegetables

Our results found that the average level of Cd in 2465 vegetables was 0.035 mg kg^−1^ wet weight (ww) (<LOD~1.82 mg kg^−1^ ww), with the P97.5 at 0.074 mg kg^−1^ ww. According to the Chinese national food safety standard GB2762-2021, the maximum allowable concentration (MAC) of cadmium is 0.2 mg kg^−1^ ww for *Apium graveolens* L., 0.1 mg kg^−1^ ww for legume vegetables, root and tuber vegetables and stem vegetables (excluding *Apium graveolens* L.), 0.2 mg kg^−1^ ww for leaf vegetables, and 0.5 mg kg^−1^ ww for other fresh vegetables. Our data showed that 3.89% (96/2465) of the samples exceeded the MAC for Cd (see Table 1). The ratio was higher than the value in our previous report, which was 0.25% in 1196 vegetable samples collected in 2016 [16], but lower than the result in another one of our reports, which was 4.37% in 343 samples collected in 2014 [15]. The data also showed that the main vegetable categories that exceeded the MAC were bulb vegetables, root and tuber vegetables and aquatic vegetables.

**Figure 2:**
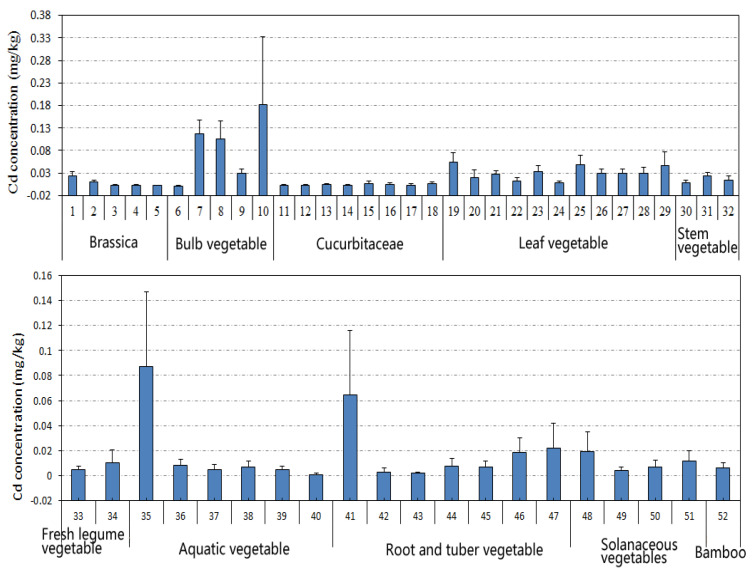
Cd levels in different vegetables. (1) *Brassica campestris* L., (2) *broccoli*, (3) cabbage, (4) cauliflower, (5) purple cabbage, (6) *Allium cepa* L., (7) *Allium chinense* G, (8) *Allium macrostemon* Bunge, (9) *Allium tuberosum* Rottler, (10) *Lilium brownii* F, (11) *Benincasa hispida*, (12) *Cucumis sativus* L., (13) *Cucurbita moschata*, (14) *Cucurbita pepo*, (15) *Lagenaria siceraria*, (16) *Lagenaria siceraria var.* hispida, (17) *Luffa aegyptiaca* Miller, (18) *Momordica charantia* L., (19) *Amaranthus tricolor* L., (20) *Apium graveolens* L., (21) *Brassica campestris* L., (22) *Brassica rapa var.* glabra RegeL., (23) *Coriandrum sativum* L., (24) *Glebionis coronaria*, (25) *Houttuynia cordata*, (26) *Ipomoea aquatica* Forsk, (27) *Lactuca sativa* L., (28) *Lactuca sativa var* longifoliaf, (29) *Spinacia oleracea* L., (30) *Asparagus officinalis* L., (31) *Apium graveolens* L., (32) *Lactuca sativa var* angustana, (33) *Phaseolus vulgaris* L., (34) *Vigna unguiculata,* (35) *Artemisia selengensis* Turcz, (36) *Eleocharis dulcis*, (37) lotus root, (38) *Oenanthe javanica*, (39) *Trapa bispinosa* Roxb, (40) *Zizania latifolia*, (41) *Colocasia esculenta*, (42) *Daucus carota*, (43) *Dioscorea polystachya*, (44) *Ipomoea batatas*, (45) *Raphanus sativus* L., (46) *Solanum tuberosum* L., (47) *Zingiber officinale* Roscoe, (48) *Capsicum annuum* L., (49) green pepper, (50) *Solanum lycopersicum* L., (51) *Solanum melongena* L., (52) Bamboo shoot.

A detailed description of the levels of 10 types of vegetables, including 52 species, is shown in Figure 2. The top 10 species with the highest average Cd concentration were *Lilium brownii* F. (0.182 mg kg^−1^ ww) > *Allium chinense* G. (0.117 mg kg^−1^ ww) > *Allium macrostemon* Bunge (0.105 mg kg^−1^ ww) > *Colocasia esculenta* (0.064 mg kg^−1^ ww) > *Amaranthus tricolor* L. (0.054 mg kg^−1^ ww) > *Houttuynia cordata* (0.0.049 mg kg^−1^ ww) > *Spinacia oleracea* L. (0.047 mg kg^−1^ ww) > *Coriandrum sativum* L. (0.034 mg kg^−1^ ww) > *Ipomoea aquatica* Forsk (0.030 mg kg^−1^ ww) > *Lactuca sativa* L. (0.029 mg kg^−1^ ww). The descending order of Cd accumulation in different types was bulb vegetable > leaf vegetable > stem vegetable > root and tuber vegetable > solanaceous vegetable > bamboo shoot > aquatic vegetable > brassica > fresh legume vegetable > cucurbitaceae. Another report also found that roots and leafy vegetables accumulated higher amounts of Cd than legume vegetables [21]. In addition to the possible pollution, the difference in Cd accumulation among various vegetables may depend on the transfer efficiency of Cd from roots to shoots or from leaves and stem to fruits.

The average Cd level (0.030 mg kg^−1^ ww) in this study was higher than the value (0.015 mg kg^−1^ ww) in 28 vegetable species reported in our previous survey [16]. We investigated more types of vegetables (52 species), particularly *Lilium brownii* F, *Allium Chinense* G, *Allium macrostemon* Bunge, and *Colocasia esculenta* with high Cd accumulation. Our data were similar to those collected from common planting areas, not the special and potential polluted zones [10,22]. However, in areas with possible contamination, vegetables were found with high Cd concentration. For example, a spinach sample with 0.479 mg kg^−1^ ww Cd was found in specific regional areas of the United Kingdom [23]. In an industrial area nearby Dhaka Export Processing Zones, Bangladesh, high Cd levels in 10 vegetable species with 0.33 ± 0.10 mg kg^−1^ were found, with a range of 0.18–0.49 mg kg^−1^ [24]. Hussain et al. [25] reported that the cadmium content in leafy vegetables, such as lettuce and spinach, was generally lower than in root vegetables, such as carrots and potatoes. González et al. [26] conducted a review on the Cd content in organic and conventional vegetables and found that the organic vegetables generally had lower levels of cadmium, likely due to the use of organic farming practices. Islam et al. [27] investigated the cadmium content in vegetables grown in different parts of Bangladesh and found that vegetables grown in areas with high levels of industrial pollution had much higher levels of cadmium.

Previous studies have also shown that the accumulation of cadmium (Cd) in vegetables varies significantly among different vegetable species. Lettuce, spinach, and parsley are among the most efficient accumulators of Cd, while peas, beans, and sweet corn are among the least efficient [28]. However, the efficiency of Cd accumulation also varies between cultivars within the same vegetable species. Furthermore, the location of cultivation can impact Cd accumulation in vegetables. For instance, a study in Bangladesh found that amaranth and spinach were among the highest Cd accumulators, while bottle gourd and ash gourd accumulated the lowest levels [29]. Similarly, in Pakistan, Cd accumulation varied widely among different vegetable species, with spinach and coriander accumulating high levels, while okra and eggplant accumulated relatively low levels [30]. Cd accumulation in vegetables was found to be highest in leafy vegetables such as lettuce and parsley, while root vegetables such as carrots and turnips accumulated relatively low levels of Cd [31]. These studies indicated that certain vegetable species are more prone to accumulating Cd than others, and this may have implications for the selection of vegetable crops for cultivation in areas with known or potential Cd contamination.

The maximum acceptable level of cadmium in vegetables varies depending on the country and the type of vegetable. The World Health Organization (WHO) has set a maximum acceptable level of 0.2 mg kg^−1^ for cadmium in leafy vegetables, 0.3 mg kg^−1^ for root vegetables, and 0.1 mg kg^−1^ for other vegetables [32]. The European Commission also sets maximum limits for cadmium in certain types of vegetables, including leafy vegetables, root vegetables, and tubers. For example, the maximum limit for cadmium in leafy vegetables such as spinach and lettuce is 0.2 mg kg^−1^, while the maximum limit for cadmium in root vegetables such as carrots and potatoes is 0.1 mg kg^−1^ [33]. For the Food Safety and Standards Authority of India (FSSAI), the maximum limit for cadmium in leafy vegetables such as spinach and lettuce is 0.3 mg kg^−1^, while the maximum limit for cadmium in root vegetables such as carrots and potatoes is 0.2 mg kg^−1^ [34]. In China, the maximum limits for cadmium in various vegetables are set as leafy vegetables 0.2 mg/kg, root and tuber vegetables 0.3 mg kg^−1^, and other vegetables 0.1 mg kg^−1^ [18]. These maximum acceptable levels are based on the best available scientific evidence and are designed to protect public health. However, it is still advisable to limit exposure to cadmium by consuming a varied and balanced diet that includes a variety of different types of vegetables.

### 3.2. Different Areas and Monitoring Years for Cd in Vegetables

**Figure 3:**
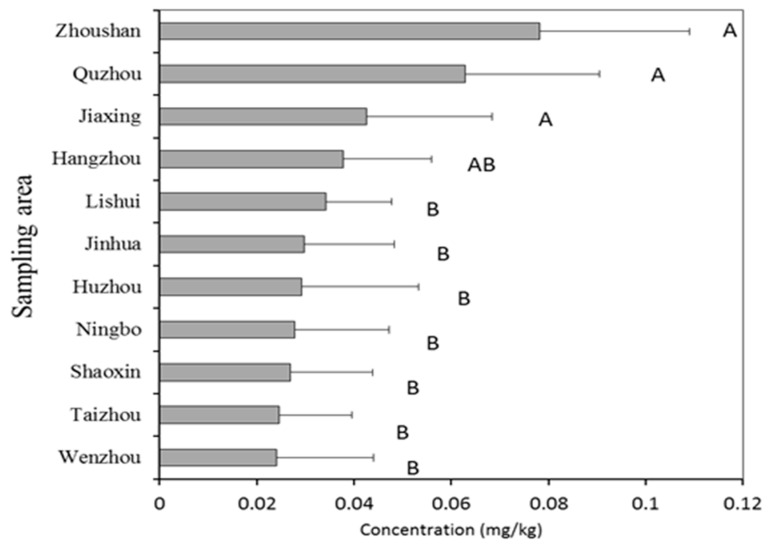
Concentrations of Cd in vegetables from different sampling areas. The columns without the same letter are significantly different at *p* < 0.05.

Geographic spatial distribution of cadmium content in vegetables was mapped based on sampling areas of 11 cities including Hangzhou, Huzhou, Jiaxing, Jinhua, Lishui, Ningbo, Quzhou, Shaoxing, Taizhou, Wenzhou, and Zhoushan. Figure 3 shows that the average content of Cd in vegetables in each region is 0.019~0.044 mg kg^−1^. The Kruskal–Wallis test was used to analyze the Cd content in different regions, and the results showed that areas of Zhoushan, Quzhou and Jiaxing had significantly different Cd content (*p* < 0.05) compared to other places (except for Hangzhou). The highest average content was found in Zhoushan, located in the northeast of Zhejiang province, and the lowest was found in Wenzhou in the southeast of Zhejiang.

**Figure 4:**
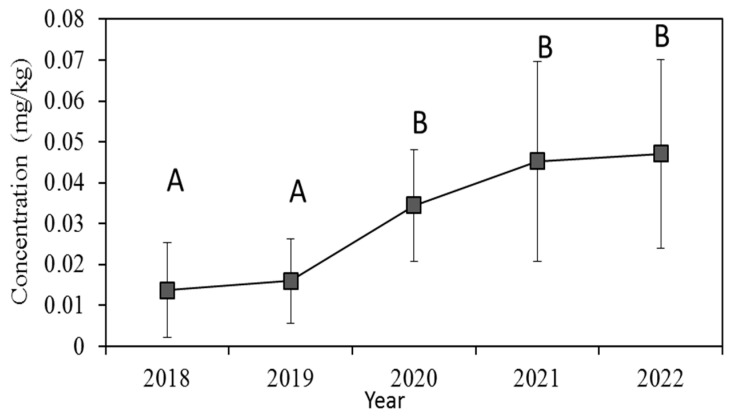
Concentrations of Cd in vegetables in different sampling years. The lines without the same letter are significantly different at *p* < 0.05.

Analysis of monitoring data from 2018 to 2022 showed a statistical difference between 2020~2019 and 2020~2022 (*p* < 0.05) (Figure 4). Over the past three years, Cd concentration in vegetables was increased compared to previous two years, which may be attributed to the varieties of vegetable species, such as bulbs, tuberous and aquatic vegetables.

The difference of Cd levels may be caused by a number of factors, including soil, cultivar variation, and atmospheric deposition [35]. The pH value of soil has been identified as playing an important role in the process of cadmium accumulation in plants [36]. Other soil properties, including organic matter, cation exchange capacity, and clay content, also affect Cd accumulation in vegetables [37]. Ouyang et al. [38] found that the concentration of atmospherically deposited Cd, rather than particle size or solubility, can regulate the foliar uptake and accumulation in water spinach *(Ipomoea aquatica* Forsk) and pak choi (*Brassica chinensis* L). Rizwan et al. [35] concluded that the uptake and accumulation of Cd in vegetables is primarily driven by two factors: (i) the concentration of Cd in the soil and (ii) the availability of nutrients such as nitrogen, phosphorus, and potassium. The study noted that Cd uptake in vegetables occurs through the roots, and that the efficiency of uptake depends on factors such as the plant species, the age of the plant, and the soil pH. Furthermore, Xia et al. reported that the Cd level varied among different pak choi cultivars based on the morphological parameters of roots [39].

### 3.3. Exposure Assessment

Exposure assessment is the process of evaluating the level of exposure of individuals or populations to a chemical, physical, or biological agent. In this study, we estimated the exposure of adults and children to cadmium in vegetables using the point estimate method. The method involves using the average value of Cd level in vegetables to represent the exposure of the general population and the P97.5 value to represent the population with high health risk.

The estimated daily intake (EDI) of cadmium through consumption of vegetables was 0.519 μg kg^−1^ bw per day for adults and 0.217 μg kg^−1^ bw per day for children in the general population (Table 2). We then calculated the Target Hazard Quotient (THQ) as the ratio of the exposure dose to a reference dose. A THQ greater than 1 indicates that the consumers are likely to experience adverse effects. In this study, THQs were lower than those reported in polluted areas. For example, Singh et al. revealed the THQ of Cd with 0.8~21 for vegetable consumption at carpet industry irrigational areas in northern India [8]. However, in southeast China, it is still necessary to pay attention to those consumers who consume large amounts of specific vegetables, such as *Lilium brownii* F, *Allium chinense* G, *Allium macrostemon* Bunge, and *Colocasia esculenta.*

Vegetables are an essential component of a healthy diet, but they can also be a significant source of Cd exposure. Health risk assessment of Cd in vegetables is, therefore, essential for the development of strategies to minimize health risks. Chen et al. [40], in China, found that the average Cd concentration in vegetables was 0.012–0.11 mg kg^−1^. The THQ values calculated for each vegetable type showed that the consumption of leaf vegetables was a risk for children, with a THQ value of 0.1, followed by tuber vegetables with a THQ value of 0.083 and fruit vegetables with a THQ value of 0.039. The study concluded that the consumption of vegetables in the region poses a significant health risk due to Cd contamination. Similarly, Wachirawongsakorn [41] in Thailand observed that the consumption of vegetables grown in Cd-contaminated soils resulted in a THQ value exceeding the safe level of 1. The implementation of strategies to reduce Cd exposure, including the use of clean soils and reducing the use of Cd-based fertilizers, was recommended. Another report by Yang et al. [42] in China found that the THQ values for Cd in vegetables were all less than 1, with the highest value observed for leek. It was suggested that the risk of Cd exposure through vegetable consumption was relatively low in the region, but individuals with high vegetable consumption may still exceed the safe limit for Cd exposure.

Cadmium is classified as a carcinogen because it has the ability to cause changes in cells that can lead to the development of cancer [43]. The mechanism by which cadmium causes cancer is not fully understood, but it is thought to involve several different processes [44]. Cadmium exposure can also lead to the activation of inflammatory pathways and chronic inflammation, which can cause damage to DNA and promote the growth of cancer cells. In addition, cadmium exposure can alter the epigenetic regulation of genes, affecting the expression of genes that control cell growth and division, and promote the uncontrolled growth of cells. Furthermore, cadmium exposure can interfere with the body’s ability to repair damaged DNA, leading to mutations in genes that regulate cell growth and division.

In this study, cancer risk analysis showed that TRs of Cd were all more than 1 × 10^−4^ for children or adults. This indicates a carcinogenic risk for exposure to Cd through the consumption of vegetables from southeast China. The CSF of Cd (6.3 mg kg^−1^ per day) used in our study was the inhalation CSF [45]. The oral CSF of Cd is not approved by the United States EPA. Furthermore, cancer involving abnormal DNA is correlated with many factors, such as environmental influence and genetic inheritance. However, there is still a potential cancer risk for the consumption of analyzed vegetables.

The uncertainty of the present assessment should be noted. The point estimation of THQ used in this study may not show the actual exposure value better than the probabilistic assessment calculated by the Monte-Carlo simulation technique [46]. The synergic or antagonistic effects for other toxic metals, such as lead, mercury, and chromium, were not considered in the assessment of health risk.

## 4. Conclusions

Our study showed that the concentration of cadmium (Cd) in vegetables from 2018 to 2022 was generally low, with no more than 5% of samples exceeding the maximum allowable concentration of Cd. However, some bulb vegetables, such as *Allium cepa* L. and *Allium chinense* G, accumulated relatively high levels of Cd. Furthermore, the study found a significant increase (*p* < 0.05) for Cd concentrations in vegetables from the years 2020 to 2022, compared to the previous years of 2018 and 2019. This upward trend in Cd concentrations could potentially increase the risk of Cd exposure and pose a threat to public health.

Health risk assessment for children and adults shows that there is low health risk associated with Cd dietary exposure for vegetables from southeast China. However, given the high levels of Cd in certain species, routine monitoring of Cd distribution in vegetables from the region is recommended. Furthermore, Cd in vegetables varied significantly across different sampling areas and years. These findings highlight the need for continued analysis and research on Cd levels in food to ensure food safety and to protect public health. Our future study will focus on more toxic elements, such as lead, mercury, and arsenic, in vegetables and investigate the possible sources of these metals.

## Figures and Tables

**Table 1:** The concentrations of Cd in vegetables (wet weight).

Types	N	Mean ^a^ (mg kg^−1^)	P97.5 (mg kg^−1^)	Range (mg kg^−1^)	MAC ^b^ (mg kg^−1^)	No. of >MAC
All vegetables	2465	0.035 ± 0.021	0.074	<LOD~1.82	-	96
Bulb vegetables	175	0.189 ± 0.105	1.20	0.008~1.82	0.05	47
Stem vegetables	177	0.010 ± 0.008	0.049	<LOD~0.099	0.1	0
Cabbage, Brassica	116	0.007 ± 0.006	0.033	<LOD~0.047	0.05	0
Solanaceous vegetables	90	0.010 ± 0.009	0.041	<LOD~0.075	0.05	2
Leaf vegetables	409	0.030 ± 0.017	0.120	<LOD~0.210	0.2	1
Fresh legume vegetables	44	0.008 ± 0.006	0.049	<LOD~0.097	0.1	0
Root and tuber vegetables	852	0.034 ± 0.022	0.124	0.006~0.740	0.1	38
Melons and vegetables (Cucurbitaceae)	256	0.004 ± 0.002	0.015	<LOD~0.041	0.05	0
Aquatic vegetables	316	0.022 ± 0.013	0.221	<LOD~0.522	0.05	8
Bamboo shoots	30	0.006 ± 0.005	0.013	<LOD~0.014	0.05	0

^a^ Target analytes with concentrations lower than LOD were treated as one-half of LOD when calculating the mean values. ^b^ MAC, the maximum allowable concentration set by Chinese government.

**Table 2:** EDI and THQ of Cd for vegetable consumption.

Consumers	EDI (μg kg^−1^ bw per day)		THQ		TR	
	Mean	P97.5	Mean	P97.5	Mean	P97.5
Adults	0.159	0.337	0.199	0.421	1.00 × 10^−3^	2.12 × 10^−3^
Children	0.217	0.458	0.271	0.573	1.37 × 10^−3^	2.89 × 10^−3^

## Data Availability

Data are unavailable due to privacy or ethical restrictions.
